# A Critical Review of the Harm-Minimisation Tools Available for Electronic Gambling

**DOI:** 10.1007/s10899-016-9624-8

**Published:** 2016-06-11

**Authors:** Andrew Harris, Mark D. Griffiths

**Affiliations:** International Gaming Research Unit, Psychology Division, Nottingham Trent University, Nottingham, UK

**Keywords:** Behavioural tracking, Breaks in play, Harm-minimisation tools, Limit-setting, Pop-up messaging, Responsible gambling

## Abstract

The increasing sophistication of gambling products afforded by electronic technologies facilitates increased accessibility to gambling, as well as encouraging rapid and continuous play. This poses several challenges from a responsible gambling perspective, in terms of facilitating player self-awareness and self-control. The same technological advancements in gambling that may facilitate a loss of control may also be used to provide responsible gambling tools and solutions to reduce gambling-related harm. Indeed, several harm-minimisation strategies have been devised that aim to facilitate self-awareness and self-control within a gambling session. Such strategies include the use of breaks in play, ‘pop-up’ messaging, limit setting, and behavioural tracking. The present paper reviews the theoretical argument underpinning the application of specific harm-minimisation tools, as well as providing one of the first critical reviews of the empirical research assessing their efficacy, in terms of influencing gambling cognitions and behaviour.

## Background

High-intensity commercial gambling has evolved relatively recently in comparison to other legalised, hazardous, and consumptive behaviours, such as tobacco and alcohol use (Adams et al. 2008). Gambling products and their advertising are now almost unavoidable and the promotion of gambling has arguably become as a social norm (Parke et al. 2014a, b). The presence of gambling has become ubiquitous, inextricably linked with national and international sporting events on television, omnipresent in towns and cities in the form of licensed betting offices, casinos, bingo halls and amusement arcades, and remote gambling, including gambling via the internet, mobile phone and interactive television (Griffiths et al. 2014).

Of particular importance is the evolution of gambling products into sophisticated, electronic platforms that possess structural features that interact with the gambler to produce ego-dystonic and maladaptive effects (see e.g., Breen and Zimmerman 2002), which may broadly be described as ‘gambling-related harm’. The strategic approach to tackling this harm is of great importance, as is the focus on efforts to reduce such harm. Adams et al. (2008) argue that in a society demonstrating relatively stable consumption, it is justifiable that attention should be directed towards the treatment of those suffering with a gambling problem. However, such concentration of effort as Adams et al. (2008) go on to argue, is less urgent in a rapidly changing environment that is demonstrating escalation of risk. Instead, effort would be best directed towards attending to the situation itself:

…when a submerged rock pierces a hole in the bottom of a boat, it makes little sense to attend solely to those who have been injured and it makes considerably more sense to focus a good deal of energy upon stemming the flow of water through the hole (Adams et al. 2008; pp. 869).

This analogy may be particularly relevant given the evolving view that the Theory of Total Consumption (Lederman 1956) is valid for gambling behaviour (Lund 2008). In the field of alcohol studies, it has long been accepted that there is a positive association between mean alcohol consumption among a population and the relative proportion of heavy or problem drinkers in that society (Babor et al. 2003). Such a relationship, originally proposed by Lederman (1956), is known as the total consumption model, or the single distribution theory. Emergent evidence suggests the total consumption model is valid in a wide variety of phenomena (Lund 2008). This has included gambling behaviour, with several studies finding evidence of increased gambling participation as gambling accessibility increases (e.g., Room et al. 1999; Turner et al. 1999), with such evidence being taken as support for the application of the theory of total consumption to gambling.

One assumption of the theory is that when individuals along the entire consumption continuum increase their gambling, this will also include those gambling at a level below or just below the limit for heavy or excessive gambling (Lund 2008). Consequently, increased gambling participation in this subgroup is enough to shift them towards the heavier gambling group. This is particularly important given the figures that demonstrate that in addition to a 0.5 % prevalence estimate for problem gambling in the UK, an additional 4.2 % of adults can be classed as ‘at-risk’ for developing a gambling problem (Wardle et al. 2014), equating to around 2.5 million people. From a total consumption perspective, increased gambling consumption has the potential to shift those at risk into the problem gambling category, as well as converting those who gamble recreationally, problem-free, to at-risk gamblers. Furthermore, for every problem gambler there are a number of family, friends and individuals in a community who are negatively impacted by problem gambling (Dickson-Swift et al. 2005) although the number of individuals affected is fewer for adolescent problem gamblers (Griffiths 1995). This provides strong argument for problem gambling to be tackled from a public health perspective.

The question remains as to how to tackle the promotion of responsible gambling (RG) and the prevention of problem gambling. This has led to the introduction of many RG and harm-minimisation initiatives. For example, one harm-minimisation approach has been to restrict the availability of gambling by reducing opening hours in licensed gambling premises (Wohl et al. 2010), as well as reduce the quantity of gambling products by restricting the number of electronic gambling machines (EGMs) in licensed betting offices in the UK to four (Association of British Bookmakers 2015). Similarly, voluntary self-exclusion programmes allow individuals who feel they have a problem with gambling to identify themselves to the gambling venue and mutually agree upon a venue exclusion for a predetermined or indefinite period of time. It is important to note that such a decision to voluntarily self-exclude may also be viewed in a positive light and from a preventive approach, as voluntary self-exclusion is available to those who may not yet have developed a gambling problem, but feel they may be at risk or simply feel like they do not want to gamble anymore.

The above examples represent the ‘supply reduction’ type of harm-minimisation. Other approaches include ‘demand reduction’, by adopting policies that make gambling less attractive, such as limiting or banning in-house smoking or the consumption of alcohol (Williams et al. 2004). Other demand reduction approaches may aim to educate customers about the true nature and odds of specific gambling games (e.g., Wohl et al. 2010), in the hope that this may enlighten gamblers that, statistically speaking, they are likely to lose money, or dispel cognitive myths relating to illusions of control or specific ‘winning’ gambling strategies, in the hope that this may reduce the desire to gamble.

The final type of harm-minimisation initiative—and the focus of the present paper—is ‘harm reduction’, which operates more from a ‘restrictivist’ philosophical and moral standpoint in tackling problem gambling. As Collins et al. (2015) identify, a restrictivist view operates somewhere in the middle of the continuum between prohibitionists and libertarianism. Unlike prohibitionists, restrictivists disagree that gambling should be banned outright, and unlike libertarians, they identify that gambling is not like any other leisure or entertainment business (Collins et al. 2015). This view argues that while gambling should be allowed, restrictions should be put in place to ensure that gambling is done so as safely and responsibly as possible.

As gambling products become more technologically sophisticated, the same technological innovation can be used to facilitate the development of harm-minimisation tools to assist gamblers in maintaining self-control and make rational and controlled gambling-related decisions. Harm-minimisation tools aim to make the time spent gambling safer, without reducing the uptake of gambling per se. Such tools have taken on a variety of forms, and while harm-minimisation as a research field within psychology is on the rise in terms of volume and quality of empirical research, the evaluation of such tools remains in its infancy. The aim of the present paper is to conduct a systematic literature review to synthesise and critically evaluate the empirical evidence available that tests the efficacy of current harm-minimisation tools. To our knowledge, while some now dated reviews have been undertaken assessing the evidence for specific harm-minimisation tools, no literature review exists that examines the collective evidence from across the harm-minimisation literature as a whole.

## Methods

### Search Strategy

An in-depth literature review was carried out comprising three concurrent phases: (1) search of online electronic databases; (2) use of professional contacts in the field of gambling to share personal collection of papers related to harm-minimisation in gambling; and (3) ‘snowballing’—a method in which reference lists from published papers are viewed and relevant papers pursued. Electronic databases included the use of the authors’ *Library One Search* (an all-encompassing database search engine—including, but not limited to: Academic Search Elite; PsychArticles; PsychInfo; Science Direct; and Scopus) as a primary source, along with *Google Scholar* being used as a more general search engine. The search terms used were *‘*gambling’, ‘gaming’, ‘electronic gambling’, and ‘online gambling’, with more specific search terms comprising ‘gambling harm-minimisation’, ‘responsible gambling’, ‘responsible gaming’, ‘pop-up messaging’, ‘responsible gambling messaging’, ‘pre-commitment’, ‘limit-setting’, ‘behavioural tracking’, and ‘gambling safeguards’.

### Inclusion Criteria

To be included as an output to be evaluated, the published paper had to have: (1) addressed harm-minimisation tools in a within-session [electronic/online] gambling context with the aim of facilitating controlled gambling (therefore, initiatives such as permanent voluntary self-exclusion schemes were not included); (2) been written in English language; (3) reported an empirical study; (4) been published within the last 10 years (2005–2015); and (5) been published in a peer-reviewed journal.

### Harm-Minimisation Tool Categorisation

Once the retrieved papers had been initially filtered according to title and abstract content, a more in-depth assessment was conducted using the inclusion criteria as guidance. The remaining papers were then categorised according to the harm-minimisation tool in question. The categories are based upon previous categorizations in the literature and are the terms most likely to be used when searching in literature databases and comprised: (1) enforced breaks in play, (2) messaging, (3) limit-setting/pre-commitment, (4) behavioural tracking tools, and (5) note acceptor prohibition or modification. These are categories that frequently appear in previous gambling harm-minimisation literature. However, it should be noted that there are several overlaps between the types of tools and the elements involved. For example, pop-up messages also contain breaks in play, and the setting of monetary limits can sometimes involve receiving a pop-up message once limits have been reached. Consequently, each tool was categorised according to its primary purpose. For example, while pop-ups provide a break in play, the message content itself is the primary harm-minimisation objective, and is therefore categorised in the ‘pop-ups’ section, and approaches assessing limit-setting with pop-up reminders when limits are reached is therefore placed in the ‘limit-setting’ sections. A summary of research findings is provided in Table 1 and overall evaluation of each tool will be given in the discussion section of this paper.

**Table 1:** Summary of included harm-minimisation studies

References	Main aims	Sample (n) (Design/method)	HM tool assessed (Game type assessed)	Main findings
*Breaks in play*				
Blaszczynski et al. (2015)	Assessed the effects of breaks in play of varying lengths in terms of their impact on cravings to continue gambling and subjective negative arousal	141 university students (78 female) (Lab-based experimental study using simulated electronic blackjack game)	Breaks in play (Electronic blackjack)	Self-reported craving higher in longer break condition. No effect of break on dissociation. Therefore, no evidence for the use of breaks in play as a way to combat dissociation was found. However, there was a significant and positive correlation between feelings of dissociation and cravings to continue play, supporting role of dissociation in continuation of gambling within a session. This effect was mediated by subjective negative arousal
*Messaging*				
Monaghan and Blaszczynski (2007)	Comparison of recall for static versus dynamic message formats.	92 undergraduate students (69 female) (Lab-based experimental study)	Static messages and pop-up messages (Electronic gaming machines)	83 vs. 15.6 % of participants were able to freely recall the message content for the dynamic and static messages respectively. Cued recall was also significantly greater for the dynamic messages (85.1 vs. 24.4 %)
Cloutier et al. (2006)	Comparison of warning messages versus pauses in play in terms of their impact on erroneous cognitions and gambling-related behaviour	40 undergraduate students (21 male) (Participants who obtained the highest scores on illusion of control questionnaire from original sample of 768. 14 participants were low-risk gamblers, 5 were at-risk gamblers, and 1 was a probable pathological gambler) (Experimental study in simulated bar setting)	Warning messages and breaks in play (Video lottery terminals)	Correcting messages, compared to pauses in play, significantly reduced erroneous thinking, but no group level effects were found in terms of the message or pause influencing gambling-related behaviour
Floyd et al. (2006)	Evaluation of warning message’s impact on gambling-related cognitions, gambling-related behaviour, as well as subjective experience during play	122 undergraduate students (70 female) (Experimental study in lab-based casino simulation)	Warning messages (Electronic roulette)	Those participants exposed to warning messages reported fewer irrational beliefs about gambling and had significantly more money remaining at the end of the session compared to participants in control condition, suggesting the messaging had some influence on subsequent gambling behaviour. Exposure to warning messages did not negatively impact on enjoyment of play
Monaghan and Blaszczynski (2010b)	Evaluated the impact of self-appraisal messaging on self-reported gambling behaviour. Such messages were compared to informative style messaging and control message conditions	Study 1, 127 regular EGM gamblersfrom university sample (male = 97) (Lab-based, experimental study) Study 2, 124 regular EGM players (male = 81) (In-vivo experimental study)	Self-appraisal messages and warning messages (Electronic gaming machines)	Both studies showed that pop-up messages were recalled more effectively than static messages immediately and at two-week follow-up. Pop-up messages reportedly had a significantly greater impact on within-session thoughts and behaviours. Messages encouraging self-appraisal resulted in significantly greater effect on self-reported thoughts and behaviours during both the experimental session and in subsequent EGM play
Harris and Parke (2015)	Experimentally assessed the impact of self-appraisal messaging on actual gambling behaviour and the interaction effect between gambling outcome and messaging efficacy	30 gamblers (18 male) from university sample reporting gambling within the last 6 months (Lab-based experimental study)	Self-appraisal messages (Electronic coin-toss)	Computer-generated self-appraisal messaging significantly reduced the average speed of betting in the loss condition only, demonstrating an interaction effect between computer-generated messaging and gambling outcome. Messages had no impact on amount wagered
Stewart and Wohl (2013)	Assessed the efficacy of monetary reminder pop-up messages in their ability to facilitate adherence to self-set monetary limits, and messaging’s impact on dissociation and craving	59 university students (43 males; 17 recreational gamblers (no DSM–IV–TR symptoms), 26 sub-threshold pathological gamblers (1–4 DSM-IV-TR symptoms), and 16 pathological gamblers (5 or more DSM-IV-TR symptoms)) (Virtual reality environment experimental study)	Monetary limit pop-ups (Virtual reality slot Machines)	Participants receiving monetary limit pop-up reminders were significantly more likely to adhere to monetary limits than participants who did not. Dissociation mediated the relationship between gambling symptomatology and adherence to monetary limits, but only among those who did not receive a monetary limit pop-up reminder. Forced stop in play created by the pop-up message did not heighten craving to continue gambling
Auer et al. (2014)	Evaluated the impact of pop-up messages in a natural and ecologically valid setting in terms of messages ability to facilitate gambling session cessation	800,000 gambling sessions (400,000 prior to pop-up being introduced and 400,000 after pop-up message had been introduced—approx. 50,000 online slot machine gamblers) (In-vivo, quasi-experimental)	Pop-up messages after predetermined number of plays (Online Slot Machine)	Found a nine-fold increase in the number of gambling session cessations at the 1000-spin mark when exposed to a pop-up message informing players of the number of plays. However, the percentage of total cessations following the pop-up message at 1000 spins was low (less than 1 %)
Celio and Lisman (2014)	Assessed the impact of a stand-alone personalised normative feedback intervention on student gambling behaviour	136 undergraduate students (75 male) reporting gambling in last 30 days (Randomised clinical trial design)	Personalised normative feedback (Self-report gambling behaviour and computer-based risk tasks)	After 1 week, those participants receiving PNF showed a marked decreased perception of other students’ gambling, as well as demonstrated lower levels of risk-taking in two analogue measures of gambling
Auer and Griffiths (2015a, b)	Evaluated efficacy of personalised normative feedback using a real world sample in a real online gambling environment. Also compared normative feedback to more simplistic pop-up messages	1.6 million gambling sessions analysed (800,000 evaluating the simple pop-up message and 800,000 evaluating the enhanced pop-up message—approx. 70,000 online slot machine gamblers) (In-vivo, quasi-experimental study)	Personalised normative feedback (Online slot machine)	Positive increase in session cessation for the more sophisticated message containing normative feedback. Only a very small percentage of sessions reached 1000 spins, meaning it is likely these pop-up messages were only given the most intense (within-session) gamblers
*Limit-setting*				
Broda et al. (2008)	Examined the effects of enforced betting limits on gambling behaviour and analysed the behaviour of those gamblers who typically exceed limits in comparison to those who adhere to monetary limits	47,000 subscribed users of the online gambling company *bwin.* (In-vivo, quasi-experimental study)	Limit-setting (Sports gambling)	Only 0.3 % of gamblers exceeded deposit limits at least once. Those gamblers who did were shown to have a higher than average number of daily bets and higher average bet sizes, compared to those who did not exceed limits. Indication that exceeding limits may be indicative of the most intense gambling sub-group
Wohl et al. (2010)	Assessed efficacy of animation-based educational video designed to facilitate adherence to pre-set limits in terms of reducing the exceeding of limits	242 non-problem gamblers (119 male) (Self-report experimental study)	Animation-based education Vvdeo (Various gambling activities)	Participants exposed to animation video reported a significant reduction in erroneous cognitions, an effect that was retained at 24-hour and 30-day follow-up. Exposure to the video also resulted in participants being more likely to strongly endorse ‘low risk’ gambling practices, including the use of limit-setting, but this effect was not retained at the 30-day follow up
Wohl et al. (2013)	Examined if there was an interaction effect between the use of educational videos dispelling erroneous cognitions and promoting safe-play, including the use of limit, and pop-up messaging reminding participants when they had reached their pre-set limit	72 young adults (51 female) with recreational gambling experience (Virtual reality environment experimental study)	Animation-based educational video and pop-up messages (Electronic gaming machines)	Participants exposed to the educational animation video adhered to pre-set limits more than those in a control video condition. Those exposed to monetary limit pop-ups also showed greater adherence to pre-set limits. These two main effects were qualified by an interaction effect, with results showing that of the participants who were not given a pop-up reminder, the ones who were exposed to the educational animation video stayed within their pre-set monetary limits more than those in a control condition
Auer and Griffiths (2013a, b)	Examined the impact of limit-setting on theoretical loss among high intensity gamblers, across a variety of gambling activities, in a real-world online setting	Random sample of 100,000 players in online gambling environment (In-vivo quasi-experimental study)	Time and Monetary Limits (Online poker, online lottery, and online casino games)	Setting limits had significant and positive effect on theoretical loss for all sub-groups of gamblers. Casino gamblers showed the biggest significant change in theoretical loss following the setting of limits
Wohl et al. (2014)	Designed new and enhanced monetary limit-setting tool using HCI and PSD principles, and compared this to older, more simple iterations of such tools in terms of their ability to facilitate limit adherence	56 current electronic gaming machine gamblers (37 female) (Virtual reality environment, experimental study)	Monetary Limit-Setting (Electronic gaming machines)	Those exposed to the HCI/PSD tool were significantly more likely to adhere to their pre-set limits compared to the standard monetary limit tool
Kim et al. (2014)	Assessed the impact of prompts encouraging the setting of time-based limits on both the uptake of setting such limits, and the impact this had on session duration	43 non-problem/low risk gamblers recruited from university sample (26 female) (Virtual reality environment experimental study)	Time limit-setting (Electronic Gaming Machines)	Participants who were prompted to set a time limit did so with a 100 % compliance rate compared to one out of 23 for those participants not prompted. Those prompted to set a limit prior to engaging in play gambled for significantly less time than those who were not prompted
*Behavioural tracking tools*				
Auer and Griffiths (2015a, b)	Assessed the effectives of the behavioural feedback system *mentor*, in terms of its ability to influence the amount of time played and theoretical loss experienced by gamblers	16,231 online gamblers (In-vivo, matched pairs, quasi-experimental design)	Behaviour Tracking and Personal Feedback (Various online gambling activities)	Online gamblers receiving personalized feedback spent significantly less time and money gambling compared to controls that did not receive personalized feedback
Wood and Wohl (2015)	Assessed the efficacy of the *PlayScan* behavioural tracking tool, which provided gamblers with behavioural feedback about their gambling, in terms of its impact on gambling behaviour	779 online gamblers (694 male) (In-vivo, matched pairs, quasi-experimental design)	Behaviour Tracking and Personal Feedback (Various online gambling activities)	At-risk players who used the feedback tool significantly reduced the amount of money deposited and wagered compared to players not utilising the tool, an effect that was obtained for both the week following enrolment and at 24-weeks later. Those gamblers who received behavioural feedback showed a significant reduction in deposited amounts compared to the control group, but this did not apply to at-risk or problematic gamblers
*Note acceptors (prohibition/lower money denomination)*				
Sharpe et al. (2005)	Tested the effects of several modifications to gaming machines, including a restriction on note acceptors to a maximum of a $20 note	779 participants of varying problem gambling severity (In-vivo quasi-experimental study)	Lower denomination note acceptor (Electronic gaming machines)	Gaming machines with modified note acceptors had no impact on any aspect of gambling behaviour compared to control machines
Hansen and Rossow (2010)	Explored the impact of prohibition of note acceptors on slot machine players in terms of its impact on gambling behaviour and problem gambling measures (SOGS-RA and Lie/Bet) in adolescent-aged gamblers	Approx. 60,000 adolescent gamblers (Questionnaire, quasi-experimental study)	Note acceptor prohibition (Slot machines)	Following prohibition, slot machine gambling frequency was reduced by 20 %, the proportion of ‘frequent’ slot machine gamblers was reduced by 26 %, and overall gambling frequency was reduced by 10 %. In addition, the proportion of problem gamblers was reduced by 20 %

## Enforced Breaks in Play

Gamblers often enter into states of dissociation (Jacobs 1986) that leads to a loss of control over time and money spent gambling. RG initiatives that temporarily stop gambling allow dissociative states to be broken and the re-evaluation of one’s gambling behaviour. Indeed, the use of enforced breaks in play as an RG tool is derived from robust theoretical underpinnings. Anderson and Brown (1984) hypothesised that arousal produced within a gambling session narrows a gambler’s attentional focus and facilitates a secondary reward of escaping psychologically distressing stimuli and wider distressing life situations. Jacobs (1986) extended this concept with his general theory of addiction, and proposed that those vulnerable to addiction were either chronically hypo-aroused or hyper-aroused. Engagement in an addictive pattern of behaviour is therefore seen as a way of maintaining homeostatic balance of arousal through generated dissociative experiences.

The use of enforced breaks in play, in the absence of supporting mechanisms such as presentation of self-appraisal messages as a RG tool (e.g., Monaghan and Blaszczynski 2010a, b), may be challenged on theoretical grounds, which indicate that breaks in play may actually have an adverse effect on the gambler. For example, the Behaviour Completion Mechanism Model (McConaghy 1980) posits that driven behaviours (includes pathological gambling), build a neuronal model of behaviour facilitated by conditioning effects. Exposure to a conditioned stimulus or cue results in the activation of the neuronal model, and any interruption to the expression of the behaviour results in an aversive state, or a state of craving, which drives the individual to the completion of the behaviour (Blaszczynski et al. 2015).

Recent research testing the efficacy of imposing short breaks in play as an RG tool challenges the use of breaks in play as a standalone RG approach. Blaszczynski et al. (2015) tested the effects of breaks in play of varying lengths in terms of their impact on cravings to continue gambling and subjective negative arousal, and compared this to a control condition featuring no break in play. Their study comprised 141 university students (78 female) who played a simulated electronic blackjack game, and were randomly assigned to an 8-, 3-min, or no break condition. Results showed that self-reported craving, as measured by the Gambling Craving Scale (Young and Wohl 2009), was significantly higher in the longer break condition, compared to the shorter break and no break condition. Significantly higher craving was also reported in the shorter break condition compared to the no break condition. It was also predicted that forcing breaks in play should reduce levels of dissociation, which has been theoretically proposed as a mechanism promoting extended play. However, no relationship between break condition and feelings of dissociation, as measured by the Dissociative Experience Scale (Jacobs 1988) was found. Therefore, no evidence for the use of breaks in play as a way to combat dissociation was found. However, there was a significant and positive correlation between feelings of dissociation and cravings to continue play, which supports the theoretical position for the role of dissociation in continuation of gambling within a session. Furthermore, the effect of the break condition on craving was mediated by levels of subjective negative arousal.

Given these findings, caution must be taken when implementing breaks in play as a standalone RG strategy. Breaks with accompanying RG messages show a certain level of positive efficacy, however, breaks alone may have unintended effects. Such effects include the promotion of cravings and desire to continue to gamble, rather than breaking dissociative states often experienced by gamblers. Conversely, limited evidence exists to give indication as to the appropriate length of break required to produce positive effects. Consequently, the efficacy of breaks should not be disregarded based on one study alone. For example, the long break condition applied in Blaszczynski et al.’s (2015) research was only 8 min long, and is open to interpretation as to whether or not this constitutes a ‘long’ break. For example, a much longer period of time may be required before maladaptive cravings dissipate and the positive effects of a break may begin to surface. However, recommendations as to what this length of time should be needs to be empirically based, but is likely to differ on an individual-by-individual basis. In addition, given differences in responses between university and real life gamblers (Gainsbury et al. 2014), it remains to be determined if the findings have external validity in terms of how such effects are applicable to real gamblers in real world gambling environments.

## Messaging

### Static Messaging Versus Dynamic Messaging

RG messages have evolved in recent times in terms of both their content and style of delivery. Originally, ‘static’ RG messages were placed at the side of gambling machines, or accessed via different menu screens on EGMs or online gambling websites (Harris and Parke 2015). This is a markedly different approach to more modern ‘dynamic messaging’ delivery systems. Dynamic messages (i.e., ‘pop-ups’) appear on-screen and deliver RG-related content whilst temporarily interrupting play (Monaghan and Blaszczynski 2007). Empirical research has demonstrated that when secondary information is delivered that interrupts a primary task, this has an orientating and focusing effect on attention that can positively impact performance on the primary task. Furthermore, this effect has been shown to last longer than the duration of presentation for the secondary information itself, indicating a sustained impact on cognitive performance (Bailey et al. 2001).

This is arguably advantageous over a static messaging approach which requires a division of attention between the primary task of gambling and processing of secondary RG information in a separate location, which may either result in messages not being salient and thus not read, or if messages are read, the information is less likely to be processed and retained due to attentional demands, which is hypothesised to be a limited resource (Broadbent 1958). Pop-up messaging in a variety of disciplines have demonstrated they have a greater impact in modifying thoughts and behaviour leading to greater task performance compared to their static counterparts (Betrancourt and Bisseret 1999).

In a gambling context, Monaghan and Blaszczynski (2007) demonstrated that message content for dynamic messages was significantly more likely to be recalled compared to static messages. In their study, 83 vs. 15.6 % of participants (92 undergraduate students) were able to freely recall the message content for the dynamic and static messages respectively. Cued recall was also significantly greater for the dynamic messages (85.1 vs. 24.4 %). Consequently, it was suggested that to maximise the effectiveness of RG messages, they should be delivered dynamically.

### Informative Messaging

While evidence suggests pop-up messaging may be an effective way to communicate RG information during a gambling session, it is important to ascertain what type of information or message should be delivered. It is also important to investigate not only if this information is processed but how effective the messages are in modifying thoughts and behaviour. Monaghan and Blaszczynski (2010a) highlighted the frequent use of educational campaigns and warning messages in public health initiatives, where the information is typically presented in the form of an indication of potential risks of specific behaviours. The ultimate goal is to moderate engagement with potentially hazardous activities and to minimise harm if individuals engage in such behaviours. It has been argued that presenting consumers with accurate information about specific products and behaviours reduces erroneous cognitions and biases, and leads to a facilitation of consumer informed choice (Monaghan and Blaszczynski 2010b).

The provision of factual information has received some empirical support in a gambling context, where the behaviour of problem gamblers has been demonstrated to be moderated by correcting erroneous cognitions, misconceptions or probability, and likelihood of winning (Ladouceur et al. 2003). Such evidence of informative messaging impacting upon behaviour is scarce in the gambling literature and indeed other health behaviour literature, including tobacco and alcohol consumption (Hammond et al. 2006). While providing gamblers with informative content may draw attention to the nature, odds, and risks involved in gambling, it has been argued that such information is relatively ineffective in modifying actual gambling behaviour (e.g., Hing 2004), although there are dated studies demonstrating informative messaging positively impacting upon gambling-related cognitions and behaviour (see Ladouceur and Sevigny 2003; Steenberg et al. 2004; Benhsain et al. 2004). More recently, Cloutier et al. (2006) demonstrated that correcting messages, compared to pauses in play, significantly reduced erroneous thinking among a sample of 40 undergraduate students who scored high on an illusion of control questionnaire. However, no group level effects were found in terms of the message or pause influencing gambling-related behaviour.

Floyd et al. (2006) advanced the pop-up messaging research by evaluating the warning message’s impact on several measures of gambling-related cognitions as well as subjective experience during play. Results demonstrated that participants in the warning message group reported fewer irrational beliefs about gambling and had significantly more money remaining at the end of the session, suggesting the messaging had some influence on subsequent gambling behaviour. Importantly, while participants reported reading on average 81 % of the messages, this did not appear to negatively impact the experience of play. Unfortunately, it is unknown whether the impact on cognitions and behaviour was facilitated by the messaging or breaks in play because there was no break condition without the inclusion of a message, so the mechanisms of change remain largely unclear. In addition, while participants in the pop-up message condition had significantly more credits remaining at the end of the session, the level of risk or frequency of bets did not differ significantly across experimental groups, making it unclear as to how a perceived increase in self-control was achieved. Furthermore, the frequency of pop-up message exposure appears particularly intrusive (despite participants not reporting a significant impact on experience of play) and unrealistic, with exposure to a message occurring every six spins.

Despite some positive results, it appears evidence for the impact of informative messaging on cognition and gambling behaviour is largely inconsistent and limited. Drawing conclusions from the existing empirical literature, it may be argued that such informative messaging has a more consistent impact on correcting erroneous cognitions, but that this effect alone is not strong enough to exert influence over gambling behaviour. However, this does not negate the use of pop-up messaging as a harm-minimisation strategy as some effect (albeit weak) appears to occur. Instead, the message content itself may be manipulated to exert a greater effect in promoting RG behaviour. Therefore, the way in which information is presented, and in turn, perceived, may be critical for its influence over behaviour.

### Self-Appraisal Messaging

Monaghan and Blaszczynski (2010a) argued that “interventions successful in improving participants’ statistical understanding of gambling do not result in any changes to gambling behaviour” (p. 71). As a potentially effective alternative, they suggested that delivering messages that directly encourage a player to self-appraise the time and money spent gambling within a session, rather than simply describing probabilities, may cause them to evaluate their behaviour in a more personally relevant manner, resulting in more considered and informed decisions relating to their gambling.

Autonomy is regarded as fundamental psychological need for the maintenance of wellbeing and positive psychological functioning (Parke et al. 2014a, b). In support of this notion, Deci and Ryan’s (1985, 2000) Self-Determination Theory argues that individuals have a fundamental need to engage in behaviour that is derived via their own value system and beliefs, rather than their behaviour being dictated from external influences. Consequently, more value is likely to be attributed to messaging that is not overly paternalistic, intrusive, and does not run contrary to an individual’s belief and value system. Pavey and Sparks (2010) argue that messages supporting an individual’s right to autonomy will be met with a less dismissive and defensive attitude.

The argument made by Monaghan and Blaszczynski (2010a) for the use of messaging that engages an individual in self-appraisal supports an autonomy-centred framework, as not only are the messages personally relevant, but also the actions taken following processing of the message will be derived through engagement with the individuals own thoughts, reflections, and motivations. This proposition for using self-appraisal messaging also has good face validity, particularly when considering the factors that contribute to problem gambling behaviour. Gamblers are often reported as experiencing dissociation from reality and absorption in the gambling task during gambling, which results in losing track of time and the experience of feelings of being outside of oneself (Monaghan 2009). Gamblers also appear to be slower to respond to external stimuli and dissociate from previous thoughts and moods (Diskin and Hodgins 1999). This overall lack of self-awareness can cause players to act in ways not previously intended, such as chasing losses and spending more money and time than they can afford (Harris and Parke 2015). RG initiatives aimed at increasing self-awareness thus appear to be a useful approach in combatting and preventing problem gambling behaviours.

Consequently, the use of self-appraisal pop-up messages as a harm-minimisation tool has received increased attention in recent years and has received some positive but limited empirical support. In a laboratory-based computer-simulated gambling experiment, Monaghan and Blaszczynski (2010b) had participants play an EGM with exposure to messages encouraging self-appraisal of time and monetary expenditure. A self-report experimental design showed that participants reported the self-appraisal messaging as having a significant influence on their thoughts and behaviour. In addition, participants also reported that the messages made them more aware of how long they had been gambling. Overall, the views of participants provided support for the application of such messages to real gaming machines in real gambling venues, as they felt that the messages would have similar impact in such environments.

In the same study, a second experiment evaluated the impact of self-appraisal messaging on self-reported gambling behaviour. Such messages were compared to informative style messaging and control message conditions. The self-appraisal messages contained information designed to engage the participant in self-reflection, and were presented in the form of questions including: “Do you know how long you have been playing? Do you need to think about taking a break?”

In comparison to informative and control messages, results showed that self-appraisal messaging had a significantly greater self-reported effect on participants’ thoughts, behaviour, and awareness of the amount of time spent gambling. While results from the two experiments showed support for the efficacy of self-appraisal messaging in influencing thoughts and behaviour, the self-report research design prevents understanding how such messages actually influence behaviour, as the incongruences between thoughts, self-report intentions, and actual behaviour in high-risk activities are well known. For example, Nevitt and Lundak (2005) demonstrated that self-report accounts of drinking habits for alcohol-offenders significantly underreported both drinking severity and the problems caused by drinking.

Harris and Parke (2015) experimentally assessed the impact of self-appraisal messaging on actual gambling behaviour. Participant’s pre- and post-pop-up exposure gambling speed of play and level of risk was measured, and by combining the two variables, betting intensity [i.e., average speed of play (bets per minute) × average stake size] was also measured. In addition, this was the first study to assess the interaction effect between gambling outcome (wins/losses) and the impact of harm-minimisation tools on gambling behaviour. Thirty participants took part in a repeated-measures experiment and were exposed to a pop-up message after 16 wagering rounds on a computer-simulated coin-toss, in both a manipulated winning and losing outcome condition, separated by a minimum of 24 h. The message simultaneously contained both instructive and self-appraisal content: “Play Responsibly…Pause and Think, Are you in Control of your Risk-Taking?”

Results showed that there was an interaction effect between messaging efficacy and gambling outcome. In the losing outcome condition, the message significantly reduced participant speed of play as measured by bets-per-minute. However, no such effect was found in the winning outcome condition, and the pop-up message failed to reduce the average wager regardless of outcome condition. In fact, average stake size continued to increase following exposure to the message. However, several limitations exist, most notably the fact that participants gambled with tokens rather than their own money. Despite the fact there was a monetary prize for the participant with the most tokens at the end of the experiment, not gambling with one’s own money is likely to have muted the effects of both the wins and losses, as well as the impact of the pop-up message in both outcome conditions. In addition, the computer-computer simulated and laboratory-based conditions did not have ecological validity and did not replicate many of the structural and situational factors associated with in vivo electronic gambling. However, this is often the trade-off associated with experiments requiring high levels of experimental control. In addition, the study was unable to identify which part of the message actually exerted a behavioural influence in terms of speed of gambling. It is not clear whether the instructive part of the message, the self-appraisal content, or indeed both parts of the message, had the impact.

### Monetary and Time-Based Pop-up Messaging

Engaging in potentially addictive behaviours, including gambling, is associated with losing track of both time and space through a process of dissociation (Jacobs 1988), particularly among problem gamblers (Diskin and Hodgins 1999, 2001; Griffiths et al. 2006). Dissociation is one potential mechanism believed to explain why many gamblers, especially problem gamblers, exceed predetermined time monetary limits (Stewart and Wohl 2013). Similarly to self-appraisal messaging, it has been argued that time and monetary pop-up reminders may combat such dissociative states as well as the failure to adhere to pre-set time and monetary limits. Stewart and Wohl (2013) conducted a randomised controlled experiment assessing the efficacy of monetary reminder pop-up messages in their ability to facilitate adherence to self-set monetary limits. University students (N = 59) with varying pre-screened levels of problem gambling severity participated in a virtual reality slot machine simulation. In support of the use of monetary pop-up reminders, results showed participants in the pop-up message condition were significantly more likely to stick to their pre-set limit (89.66 %) compared to a control (no pop-up) condition (43.33 %). Results also showed that higher gambling symptomology and dissociation were associated with lower monetary limit adherence. The fact that there was no mediating effect of dissociation on limit adherence in the pop-up condition (but was found in the control condition) led the authors to suggest that the presence of the pop-up stopped participants experiencing dissociation.

Auer et al. (2014) conducted the first ever study evaluating the impact of pop-up messages in a natural and ecologically valid setting. More specifically they examined whether a pop-up message presented after 1000 consecutive plays of an online slot machine would help players cease their gambling. The pop-up message simply informed players: “You have now played 1000 slot games. Do you want to continue? (YES/NO).” The 1000-spin mark was chosen as this equated to approximately 1 h of play, and empirical evidence suggests that this is a key point in play where pop-ups may be most effective (see Ladouceur and Sevigny 2009). The authors’ analysed 800,000 online slot machine gambling sessions, comprising of approximately 50,000 gamblers. Data sampled from 400,000 sessions prior to the introduction of a pop-up message showed that of the 4220 games that consisted of 1000 or more consecutive slot machine spins by the same players, only five sessions ended at 1000 spins. A further 400,000 sessions were analysed after the introduction of the pop-up message. Of these 400,000 sessions, 4205 contained at least 1000 consecutive slot spins, which were then in turn exposed to the pop-up message. Forty-five of these sessions were terminated following pop-up exposure.

While the data set was too large for inferential statistics to be applied, results showed a nine-fold increase in the number of gambling session cessations at the 1000-spin mark when exposed to a pop-up message reminding players of the length of their play. However, despite this increase, the percentage of total cessations following the pop-up at 1000 spins was still very low (<1 %). It is important to note that of the 800,000 total sessions analysed, only a very small number (approximately 1 % of all session), reached 1000 consecutive spins by the same player, indicating that the study largely dealt with the most gambling-intense individuals. This finding has a number of potential implications. Firstly, it may be better to introduce pop-ups at an earlier stage of play to capture a larger sample of gamblers. Secondly, the results of the study indicate the relative ineffectiveness of such pop-up interventions for most (within-session) gambling-intense individuals.

### Normative Feedback and Enhanced Messaging

The use of normative feedback, delivered via the platform of a pop-up message, is a potential way to facilitate behavioural change, and is beginning to receive attention in the gambling literature. Personalised normative feedback (PNF) aims to correct an individual’s perception about the normal levels of engagement in specific behaviours by others. Normative feedback has been shown to have an influence on a variety of potentially hazardous behaviours, including smoking, where PNF increased smoking cessation (Van den Putte et al. 2009), increased condom use (Yzer et al. 2000), and reduced marijuana consumption (Yzer et al. 2007). The use of PNF also has clinical utility, where it has been shown to be important when incorporated into motivational interviewing (Miller and Rollnick 1991).

The application of PNF in a gambling context has also received some empirical support, where it has been shown to exert both perceptual and behavioural influence.^1^ Celio and Lisman (2014) assessed the impact of a stand-alone PNF intervention on student gambling behaviour. Undergraduate students (N = 136; 55 % male) who reported gambling in the past 30 days were recruited to take part in a randomised clinical trial design. Participants were assigned to receive either PNF or an attention control task. In addition to self-report, Celio and Lisman’s (2014) study used two computer-based risk tasks framed as “gambling opportunities” to assess cognitive and behavioural change at 1 week post-intervention. Results showed that after 1 week, those participants receiving PNF showed a marked decreased perception of other students’ gambling, as well as demonstrated lower levels of risk-taking in two analogue measures of gambling.

Auer and Griffiths (2015a, b) extended the validity of the use of PNF as an RG tool by evaluating its efficacy using a real world sample in a real online gambling environment. Furthermore, the research design compared the efficacy of PNF pop-up messages (in combination with additional message content) to more simplistic forms of pop-up messages. The simplistic message (as outlined above in their previous pop-up message study) was enhanced and read:

We would like to inform you that you have just played 1000 slot games. Only a few people play more than 1000 slot games. The chances of winning does not increase with the duration of session. Taking a break often helps, and you can choose the duration of the break (see Auer and Griffiths 2015a, b, p. 3).

A total of 1.6 million gambling sessions were analysed (800,000 evaluating the simple pop-up message and 800,000 evaluating the enhanced pop-up message). In the simple pop-up condition, 11,232 sessions lasted at least 1000 spins and these players were exposed to the pop-up (1.4 % of the total sessions). Of the 11,232 sessions, 75 were immediately terminated following pop-up exposure (0.67 %). In the enhanced pop-up condition, 11,878 sessions lasted at least 1000 spins (1.48 % of the total sessions). Of the 11,878, 169 were immediately terminated following pop-up exposure (1.39 %). The percentage of those stopping their gambling session at 1000 spins was significantly higher for the enhanced PNF message compared to the simple message.

While this positive increase in session cessation for the more sophisticated message is promising from an RG perspective, several limitations are noted. Firstly, the enhanced message not only contained normative feedback, but also contained informative and self-appraisal content, so understanding which element or elements of the message had the most behavioural influence cannot be ascertained. Secondly (and as with their previous study), only a very small percentage of sessions reached 1000 spins, meaning it is likely these pop-up messages were only given the most intense (within-session) gamblers. Finally, the normative part of the message was only a general statement, and therefore the effects of more specific normalised feedback were not assessed.

## Limit-Setting

Gamblers frequently spend more time and money than initially intended (Monaghan and Blaszczynski 2010a). Furthermore, exceeding financial time and monetary limits within a gambling session has been identified as a key risk behaviour for the development of problem gambling. Failure to stick to pre-set limits arguably reflects a loss of, or impairment in, self-control and self-regulation, which can be undermined by a variety of factors (Parke et al. 2014a, b). Such factors include an inability to regulate emotion (Scanell et al. 2000), and the use of emotion in the decision-making process over the use of problem-focused strategy (Blaszczynski et al. 1990).

Limit-setting is a harm-minimisation strategy that allows gamblers to set time and monetary limits prior to commencement of a gambling session. Limit-setting is based on the principles that decisions concerning time and monetary limits (a) should be made in a state of non-emotional arousal, and (b) once made, must be adhered to for the remainder of the gambling session (Ladouceur et al. 2012). Limit-setting represents an RG tool designed to prevent excessive expenditure in individuals prone to impaired self-control, as well as those who wish to use the feature as a positive, pre-emptive measure. The intention of limit-setting is to promote rational decisions regarding expenditure in advance of play, and, by imposing barriers, to ensure compliance with such decisions when emotionally aroused after losses (Ladouceur et al. 2012), or indeed, wins. Evidence for its use also comes from the natural recovery literature, where it has been shown that 40–82 % of individuals with a gambling disorder recover without professional help (e.g., Abbott et al. 1999). One of the primary techniques adopted by such self-recovery populations was the use of self-imposed time and/or money limits (Blaszczynski and Nower 2010).

Setting limits on gambling time and monetary expenditure may also be viewed as a form of public commitment, and past research indicates that publicly committing to a goal will increase the chances of that goal being reached (Mussell et al. 2000). Outside of gambling, such public commitment strategies have been successfully applied in other areas of health research such as weight loss programmes (e.g., Nyer and Dellande 2010).

Broda et al. (2008) examined the effects of enforced betting limits on gambling behaviour and analysed the behaviour of those gamblers who typically exceeded limits in comparison to those who adhered to monetary limits. Two years of sports gambling behavioural data were analysed from 47,000 subscribed users of the online gambling company *bwin*. Only a very small proportion (0.3 %) exceeded deposit limits at least once. Gamblers who did were shown to have a higher than average number of daily bets and higher average bet sizes, compared to those who did not exceed limits, indicating that exceeding limits may be indicative of the most intense gambling sub-group. Furthermore, behaviour after exceeding limits showed that average bet sizes steeply increased, though the number of bets reduced. Results indicated that the setting of limits, accompanied by a reminder once limits have been reached, was enough to deter the vast majority of gamblers from exceeding those limits. However, the small majority of those who exceeded limits may represent the most heavily involved gamblers, and arguably, the most in need of help, suggesting the use of limit-setting may be best placed as a preventative RG tool, rather than an intervention for those who may already be exhibiting gambling problems.

Wohl et al. (2010) applied the principles of the Health Belief Model (HBM; Janz et al. 2002) to an animation-based educational video designed to facilitate adherence to pre-set limits. The HBM predicts that healthy and adaptive behaviour will be adopted by individuals when an intervention has a targeted and specified impact on the knowledge, attitudes, and perceptions of target group members. This was applied in a gambling context, more specifically, during slot machine gambling, where the HBM suggests that risk behaviours will be reduced if players come to understand: (1) the true odds of winning, (2) that odds do not improve with persistence. (3) that the consequences of exceeding financial limits can be serious and difficult to reverse, (4) that staying within affordable limits eliminates the chances of developing gambling problems, and (5) that low-risk practices can be used to stay within affordable limits.

A total sample of 242 non-problem gamblers were recruited. Those exposed to an educational animation video applying the principles of the HBM, designed to dispel cognitive distortion, and promote the use of and adherence to time and monetary limits, reported a significant reduction in erroneous cognitions, an effect that was retained at 24-h and 30-day follow-up. Exposure to the video also resulted in participants being more likely to strongly endorse ‘low risk’ gambling practices, including the use of limit-setting, but this effect was not retained at the 30-day follow up. In addition, the video promoted greater behavioural intention to use the ‘low-risk’ practices, but again, this effect was not retained at the 30-day follow-up. Finally, participants exposed to the video reported exceeding their self-set limits less often (8 vs. 25 % for a control group), but again, the effect was not retained at 30-day follow-up.

Clearly, the self-report method applied is subject to inaccuracies, and behavioural intention does not always lead to behavioural execution, particularly in situations where demand characteristics may be working to provide positive outcomes. Alternatively, the effects of the animated video may be more subtle and not noticed by participants, meaning the failure to find a lasting effect at 30-day follow-up may simply be a failure for participants to experientially detect a change, and not necessarily portray a lack of change. What is required is empirical behavioural gambling data to measure pre-and post-intervention effects. It must also be noted that the effects of the video on cognitive distortions were long-lasting, which may equip individuals well in the long run as a protective factor against developing problems with gambling, but longitudinal evidence is required to test such a proposition.

Using a virtual reality gambling environment, Wohl et al. (2013) examined if there was an interaction effect between the use of educational videos dispelling erroneous cognitions and promoting safe-play, including the use of limit-setting, and pop-up messaging reminding participants when they had reached their pre-set limit. Participants were 72 young adults (mean age = 19.69 years, SD = 1.82) with recreational gambling experience, and were predominantly female (70.8 %). Participants played an EGM in a virtual reality environment, gambling with a total of 80 credits ($20). Results showed participants exposed to the educational animation video adhered to pre-set limits more than those in a control video condition (97 vs. 77 %). Those exposed to monetary limit pop-ups also showed greater adherence to pre-set limits (97 vs. 77 %). However, these two main effects were qualified by an interaction effect, with results showing that of the participants who were not given a pop-up reminder, the ones who were exposed to the educational animation video stayed within their pre-set monetary limits more than those in a control condition (94.1 vs. 61.1 %). However, no difference was found in limit-adherence among the participants who all received monetary pop-up reminders, but either saw or did not see the education animation video. The authors concluded that from an RG perspective, there was no additive effect of exposure to both RG tools, and thus, pop-up messages reminding gamblers when they have reached their pre-set limits would be the most effective and efficient RG tool.

It should also be noted that only the education video had a significant effect on reducing erroneous cognitions, and in the absence of pop-up messages, exposure to the video had an effect on gambling behaviour in terms of limit adherence. This shows the potential for education animations as an RG tool, but that it may not be as effective as other measure such as pop-ups in terms of their efficacy in influencing gambling behaviour during play. There is potential for strategies such as educational animations, or education in general, to be applied where pop-ups may not be feasible, for example, in literature in and around gambling venues, or as part of a mathematics curriculum in schools. However, the effect on problem gamblers remains unknown.

Auer and Griffiths (2013a, b) examined the efficacy of limit-setting among high intensity gamblers, across a variety of gambling activities, in a real-world online setting. Data were initially collected from a representative random sample of 100,000 players, of which 5000 had opted to use the voluntary time and/or monetary limits. The top 10 % most intense gamblers, as derived via theoretical loss (house advantage multiplied by amount wagered; see Auer et al. 2012), were taken from each of the sub-gambling type groups (i.e., poker, lottery, and casino games). Results showed that theoretical loss significantly decreased among the top 10 % most gaming-intense lottery players in the 30 days following all kinds of voluntary limit-setting (time and money) compared to the total theoretical loss in the 30 days prior to the implementation of limits. The impact of the cash-in limits on theoretical loss was higher than playing duration limits. Similarly, limit-setting decreased the theoretical loss for the top 10 % most intense casino gamblers. However, time limits had no significant impact on theoretical loss for this subgroup. It was also noted that casino gamblers showed the biggest significant change among the general gambling population, with 77 % of the theoretical loss being spent in the 30 days following limit-setting compared to theoretical loss in the prior 30 days. Among the top 10 % most intense poker players, the amount lost in the poker rake decreased in the 30 days following limit-setting, but this was only the case for those who set weekly spend limits and daily time limits. Overall, time limits had the greatest effect on rake loss for poker players, with those setting daily time limits losing 73 % of the loss in the 30 days prior to the setting of limits. As expected, the setting of daily time and session length limits had a highly significant effect on overall play duration. This is important given the fact that excessive time spent gambling, and not just excessive monetary spent, can have deleterious impacts on the lives of gamblers.

The behavioural tracking paradigm used in this study only gives information about gamblers on one particular gambling site and does not identify the overall profile and behaviour of a particular gambler. This is important as the most problematic gamblers have been shown to play multiple types of gambling platforms concurrently (McCormack et al. 2013), which may mean that reaching monetary or time limits on one site, on one platform, does not necessarily mean cessation of gambling until such limits are reset. It may simply mean that gamblers switch from one site to another once a self-set limit has been exhausted. Pairing (or grouping) of online gambling accounts may be a way around this issue, much like the facility afforded by gaming operators such as *Pokerstars* and *Full Tilt*. Of course, this relies on cooperation among competing gambling operators to be a viable option, but it would allow the potential for ‘central’ limits to be set across all of an individual gambling accounts, rather than several isolate limits set at each of the sites where and gambler has an account.

The focus on the most intense gamblers is certainly of relevance given the fact that this sub-group is most likely to benefit from limit-setting. However, the results provided do not tell us how the majority of gamblers, falling more centrally in the distribution curve, interact with limit-setting. As limit-setting is often viewed as an RG tool with preventive utility (see Wohl et al. 2014), such large scale, real-world, behavioural tracking techniques should also be applied to those gamblers below the threshold for problem gambling criteria.

Using the principles of Human Computer Interaction (HCI) and Persuasive System Design (PSD), Wohl et al. (2014) aimed to improve the efficacy of monetary limit-setting as an RG tool, by improving the way that gamblers interact with such features in electronic gambling. HCI principles suggest that for technology to be user-centred, potential users must be involved in the design, testing, and evaluation process. Consequently, they conducted a series of focus groups involving non-problem gamblers discussing their views one existing limit-setting tools, as well as discussing potential design improvements that may increase the tools RG utility.

Using information gained from the focus groups, Wohl et al. then designed new monetary limit-setting with pop-up message reminder, and compared this to older, more simple iterations of such a design. New monetary reminder pop-up message features included a traffic light visual display, informing participants of their spend relative to their limits (i.e., green light ‘safe’, amber ‘close’, red light ‘limit reached’), this was to allow self-monitoring of behaviour, one of the principles of PSD. Once limits had been reached, a 1-min delay was enforced before players could opt to continue to play. Fifty-six EGM gamblers (37 females) were recruited and participated in an EGM simulation in a virtual reality environment. They gambled with 80 credits ($20) and any money left at the end of experiment was kept by the participant. Gambling outcome was controlled for by the experimenter to ensure all participants reached their limits.

Only seven participants (three from the HCI/PSD condition, and four from the standard monetary limit-setting condition) failed to reach their limits and were thus excluded from subsequent analysis. Results showed that those exposed to the HCI/PSD tool were significantly more likely to adhere to their pre-set limits compared to the standard monetary limit tool (62.2 vs. 2 % respectively). Also of importance was the fact that two participants stopped prior to reaching their limits immediately after viewing their player statistics. Self-report data also indicated that participants perceived more engagement with the HCI/PSD tool. However, encouragingly, mean ratings for both the HCI/PSD and old design were above the mid-way point of the scale, showing perceived engagement in both conditions.

Using an EGM simulator in a virtual reality environment, Kim et al. (2014) assessed the impact of prompts encouraging the setting of time-based limits on both the uptake of setting such limits, and the impact this had on session duration. Forty-three, non-problem/low risk Canadian university student gamblers were recruited and given $20 to gamble with in the experiment. Analysis revealed that participants who were explicitly asked to set a time limit did so with a 100 % compliance rate (20/20), compared to just one out of 23 for those participants not prompted to set limits. Those prompted to set a limit prior to engaging in play gambled for significantly less time than those who were not asked to set a limit (5 vs. 9.48 min respectively). Of note, 11 out of 20 of participants in the limit-setting group gambled for less time than their self-set limit.

Several limitations exist, including then potential for demand characteristics in the experimental paradigm to drive the high percentage of participants setting limits. In such a laboratory environment, many structural and situational characteristics of real gambling environments are lacking, all of which may draw attention away from the available RG tools. In addition, participants were only exposed to a single RG tool, and thus, the study cannot report the relative additive (or deleterious) impact that multiple available tools can have in moderating gambling behaviour. However, the results indicated that setting limits on gambling session duration may be effective as an RG tool by reducing the amount of time an individual spends gambling. The authors note that while some gambling activities may benefit from the use of monetary limits, some activities may benefit from time limits. This is perhaps particularly relevant for gambling platforms such as EGMs, where there may be a tendency to dissociate and lose track of time (see Diskin and Hodgins 2001), or poker, where tournaments are typically long and cash games have no defined end as such.

## Behavioural Tracking Tools

Research indicates that providing gamblers with personalised feedback helps them to better understand their behaviour and change it if necessary (Auer and Griffiths 2013a, b). Digital technology affords the opportunity to track behavioural player data, which in turn, allows the opportunity to profile gamblers, assess behavioural change that may be indicative of a problem developing, and thus, provide gamblers with personalised feedback to facilitate awareness of such behavioural change. Behavioural tracking also produces datasets that allow identification of behavioural markers that may be indicative of harm, which in turn, further allows the development of understanding related to both responsible and problematic gambling practices.

Auer and Griffiths (2013a, b) argue that personalised messages can be applied using the principles of motivational interviewing, where behavioural tracking allows the delivery of personal, transparent, and motivational feedback. They argue that the target population for behavioural tracking tools should be those who are ‘at-risk’, or those who are developing a problem. Behavioural tracking tools may therefore provide motivation for change via the use of personalised feedback, and for this reason, personalised feedback via behavioural tracking is in line with the Stages of Change Model (SCM; Prochaska et al. 1994). The SCM has been applied to a broad range of behaviours, including weight loss and alcoholism, where the idea is that behaviour does not change in one step, rather, change occurs through a series of steps, starting from pre-contemplation, all the way through to maintenance of a behavioural change (for example, see Prochaska et al. 1994).

Auer and Griffiths (2015a, b) assessed the effectives of the behavioural feedback system *mentor*, in terms of its ability to influence the amount of time played and theoretical loss experienced by gamblers. Behavioural data were obtained from a European online gambling site, with a sample of 1015 gamblers who had used the *mentor* system. A matched pairs design was used to compare behavioural change of gamblers who opted into use the *mentor* behavioural feedback system, with behaviour of gamblers who did not use the mentor system (n = 15,216), and were matched for age, gender, playing duration, and theoretical loss in the 14 days prior to uptake of the *mentor* system for the experimental group. The *mentor* system also applied the principles of HCI and PSD (see Wohl et al. 2014), and provided players with visual feedback in the form of a graphs with the amount of time and money spent gambling in comparison to normative behaviour of other gamblers in the database. Results indicated that of the 1015 gamblers using the *mentor* system, 625 (62 %) showed a smaller theoretical loss ratio and 60 % showed a shorter playing duration ratio in comparison to theoretical loss and playing duration of matched control group ratio (12 and 10 % above chance level respectively). The findings indicated that overall, gambling behaviour of those using a personalised behavioural feedback system decreased more than control group members.

While a difference in behaviour as a consequence of the personalised feedback system was found, the effects were small, which means a degree of caution is required before a full endorsement of behavioural feedback is made. In addition, a limitation of the study includes the fact that no information about the gambler’s level of risk or problem gambling status was obtained. Consequently, it is not known whether the tool was most effective for those players with problem gambling tendencies, or whether the tool was most effective in moderating the behaviour of those gamblers who already gambled responsibly. In addition the study was unable to determine if the gamblers were concurrently using any other gambling sites or platforms during the evaluation period.

Wood and Wohl (2015) assessed the efficacy of the *PlayScan* behavioural tracking tool, which provided gamblers with behavioural feedback about their gambling, in terms of its impact on gambling behaviour. A sample of 779 gamblers (694 males) who opted into use *Playscan* was obtained from the online gambling site *Svenska Spel*. Gambling behavioural data was compared for those who opted into use the *Playscan* system with matched controls who did not opt in. The behavioural feedback utilised an algorithmic system which provides players with a colour-coded risk rating according to their expressed behaviours, with green indicating no issues, yellow being at-risk, and red being problematic. Gambling expenditure data (deposit and wager amounts) were gathered for the week in which players enrolled on *Playscan*, as well as the subsequent week and 24 weeks later. These data were also gathered for the matched pairs control group.

Results showed that at-risk players (‘yellow’ players) who used the feedback tool significantly reduced the amount of money deposited and wagered compared to players not utilising the RG tool. Furthermore, this effect was obtained for both the week following enrolment and at 24 weeks later. Results indicated that those gamblers who received behavioural feedback showed a significant reduction in deposited amounts compared to the control group in the week after enrolment. However, ‘red’ and ‘yellow’ players (i.e., those showing signs of problematic or risky play) did not significantly reduce their deposit amounts in this period compared to a control group. Only the ‘green’ group showed a significant deposit reduction for this period, relative to the control group. However, deposit reductions were noticeable over time, with ‘green’ and ‘yellow’ gamblers showing a significant deposit reduction from week of enrolment to week 24 compared to the control group. There was no such reduction over this period of time for red players.

In terms of wagering amounts, while ‘red’ players reduced their wagering between enrolment and 24 weeks later, this amount did not differ compared to the control group. However, for the same period, ‘yellow’ and ‘green’ gamblers significantly reduced their wagering amounts compared to a control group. This suggests that behavioural feedback via behavioural tracking may have a positive impact in keeping controlled gamblers safe, as well as positively impacting at-risk players, while the effects on those gamblers already exhibiting problematic symptoms appears minimal. This supports the notion of behavioural feedback as an RG tool aimed at preventative measures, rather than an intervention for problem gamblers. However, as the authors noted, the extent to which the colour classifications actually relate to more standardised measures of problem gambling is unknown.

## Prohibition and Modification of Note Acceptors

One method that had been implemented in Norway as a way to reduce gambling expenditure and gambling-related harm is the prohibition of note acceptors on slot machine, which produced a 40 % reduction in the turnover produced by slot machines (see Norwegian Gaming Authority 2006, 2007). The prohibition or restriction of note acceptors appears to be a valid avenue of exploration in RG, particularly given evidence suggesting problem gamblers more frequently use high denomination bank notes when gambling compared to non-problem gamblers (Sharpe et al. 2005). Despite evidence from Australia that (1) suggests problem gamblers prefer to use note acceptors while gambling (Australian Productivity Commission 1999), and (2) there is a strong correlation between problem gambling and use of note acceptors (McMillen et al. 2004), there is very little empirical evidence demonstrating the efficacy of prohibition/restriction of note acceptors in reducing problem gambling among EGM players.

Sharpe et al. (2005) tested the effects of several modifications to gaming machines, including a restriction on note acceptors to allow a maximum of a $20 note. The research was carried out in an ecologically valid environment, with 779 participants of varying problem gambling severity playing on the modified gaming machines in hotels and bars. Several proxy measures of gambling behaviour recorded, including time spent gambling, number of bets, net loss, and lines per wager. However, machines with restrictions on note acceptors failed to have any significant impact on any aspect of gambling behaviour compared to control machines.

The authors highlighted several limitations of the research, including the fact that a large proportion of gamblers approached to take part in the study declined, bringing into question how representative their sample was. Other limitations included the potential part that demand characteristics played on participant gambling behaviour, due to the fact that participants were being observed by the experimenter to record gambling behaviour. In addition, there were an insufficient number of probable problem gamblers in the sample to compare whether the machine modifications had differential efficacy in modifying behaviour for problem gamblers in comparison to non-problem gamblers.

Hansen and Rossow (2010) explored the impact of prohibition of note acceptors on slot machine players in terms of its impact on gambling behaviour and problem gambling measures (SOGS-RA and Lie/Bet) in adolescent-aged gamblers. The samples comprised 20,703 students in 2004 (pre-intervention); 21,295 in 2005 (pre-intervention); and 20,695 in 2006 (post-intervention). Respondents were mostly 13–19 years old with an average age of 15 years and there was an approximate 50/50 gender split. Importantly for the efficacy of note acceptor prohibition as an RG measure, results showed no significant changes in gambling behaviour and problem gambling at time points one and two (pre-prohibition). However, significant differences were found at time point three following prohibition. Following prohibition, and controlling for potential confounding variables, slot machine gambling frequency was reduced by 20 %, the proportion of ‘frequent’ slot machine gamblers was reduced by 26 %, and overall gambling frequency was reduced by 10 %. In addition, the proportion of problem gamblers was reduced by 20 %. No significant gender differences were found.

Only one-third of adolescent gamblers reported noticing the removal of bank note acceptors, and two-thirds reported either stopping gambling or reduced gambling following the prohibition. Hansen and Rossow (2010) reported that only a small fraction of participants attributed the changes in their gambling behaviour to the removal of bank note acceptors. Importantly, no compensatory behaviour in terms of transition to other forms of gambling was observed after the intervention, and decreases in gambling behaviour were also observed for both at-risk and problem gamblers.

A limitation of the research is that it does not offer explanatory value in terms of the mechanisms of change. One argument proposed by Hansen and Rossow (2010) stated that an inability to use notes slows down the speed of play, where speed of play has frequently been implemented as a problematic characteristic of electronic gambling (McCormack et al. 2013). In addition, it is possible that the need to transfer notes into coins may break up the rhythm of play, which may have the added effect of breaking dissociative states and raising levels of self-consciousness regarding gambling time and monetary expenditure. The time taken to transfer notes to coins, or the associated increased time it takes to load a machine with coins, may be sufficient time to allow any increased levels in stress and arousal to dissipate, allowing gambling decisions to be made rationally in a ‘cold’ (as opposed to ‘hot’) emotional state (Parke et al. 2014a, b).

## Discussion

It is now widely accepted that delivering RG information during play, to facilitate self-awareness, self-control, and dispel erroneous cognitions, should be delivered via a dynamic mode of display using pop-up messaging. In terms of messaging content, despite some positive results, evidence shows an inconsistent effect of informative style message content on gambling behaviour. Informative content aimed at dispelling cognitive biases and erroneous cognitions related to gambling appear to be more effective. However, such an effect appears to inconsistently transfer to gambling-related behaviour. Such research also suffers from the limitation that it is often unclear as to whether it is the message content itself, or the break in play offered by the message, that exerts the behavioural influence. Recent evidence shows there can be adverse impacts using breaks in play in isolation of RG messages on cravings and negative valence (Blaszczynski et al. 2015). This suggests that it is not the break in play afforded by pop-ups in the pop-up literature that facilitates behavioural change. However, it cannot be established if the two in combination provide an additive effect.

As a consequence of the relative inconsistencies of informative messaging on gambling behaviour, other approaches, such as the use of self-appraisal messaging, normative feedback, and the use of time and monetary reminders have begun to be explored with often significant results but small effect sizes. These studies represent a diverse methodological approach, encompassing self-report, experimental laboratory work, and ecologically valid experimentation that offsets the weaknesses of each approach used in isolation. However, current research carried out in real world environments appears to have a focus on the most intense gamblers, and while significant results in the intended direction have been found, particularly in terms of messaging facilitating gambling cessation, the effects are small, and do not tell us anything about the influence of messaging on the majority of gamblers who gamble at moderate and safe levels.

Counter to this argument is the fact that the most intense gamblers are likely to be the ones most in need of help to remain in control, and if messaging is able to help only small numbers of gamblers, then this should be regarded as positive (given that the mantra of many gaming operators is that “one problem gambler is one problem gambler too many”). However, RG tools should strive to assist more than a few gamblers, and pop-up messaging may be regarded as a preventative tool rather than an intervention for problem gambling. Consequently, longitudinal research may be of value to evaluate the relative effectiveness of messaging in term of helping the majority of gamblers, and those gambling recreationally, to stay in control.

While significant findings in the intended direction for pop-up messaging are emerging, it is suggested that researchers and the gaming industry should not be content with the results, and that research also needs to remain flexible and continue to explore the impact of other approaches to messaging content, both in isolation and in combination with other forms of messaging content. For example, it could be that the use of emotional imagery, emotion-laden content, and self-set messages offer a potentially successful alternative to current approaches. Implementation of such new approaches should continue to evolve from controlled laboratory-based investigations to real-world testing before widely implemented, as well as being tested on the diverse sub-groups of gamblers covering the entire spectrum of gambling behaviours, ranging from recreational through to pathological.

Combined, empirical data from both laboratory-based and real-world environments has shown positive results for the use of limit-setting as an RG tool. However, limit-setting research does not address the issues of gamblers being able to switch gambling platforms once limits have been reached. Other methodological limitations, such as the failure to account for concurrent gambling expenditure outside of the boundaries of the studies of focus, makes it hard to make any conclusive statement about the overall effectiveness of limit-setting as a harm-minimisation tool. Furthermore, in EGM play, limits can be set, reached, and then overridden with the continuation of play when gamblers may be in elevated states of arousal and experiencing negative emotion, albeit following a brief pause in play.

Currently, limit-setting is not mandatory in most countries. A mandatory limit-setting system, such as that in Norway, has the advantage of helping both recreational and problem gamblers adhere to pre-set limits and assists them in avoiding loss chasing, but this does not avoid the issue of gamblers potentially switching gambling platforms, although how often this occurs is yet to be established (Parke et al. 2014a, b). A voluntary limit-setting system does have some advantageous qualities over mandatory limit-setting, in the light of Self-Determination Theory (Deci and Ryan 1985, Deci and Ryan 2000), in the sense that the free choice to self-set limits will more likely result in behavioural execution of limit adherence, as well as instil a more positive attitude towards the tool more generally, given the fact that decisions will be derived through the gambler’s own value system and motivations. This does not address the potential transition from a pre-session gambler, operating in a ‘cold’ emotional state, making rational decisions, to one who may be experiencing negative valance following losses, in a highly aroused state, making emotion-based choices, where reaching their pre-determined limit can be easily overridden following a pop-up reminder. Of course some sites, such as *Pokerstars*, enforce a much longer delay period once pre-set deposit limits have been reached, allowing a much longer ‘cooling-off’ period. What may be required for EGMs or online gambling games is for sessions to be mandatorily terminated once limits have been reached, rather than asking gamblers if they would like to continue following a reminder and short delay. Although this would not address the potential for gamblers to switch terminals to the one in their immediate vicinity, or simply move venues, it may provide the delay required for the dissipation of highly aroused and emotional states.

Encouragingly, limit-setting research has started to incorporate psychological principles founded in wider areas of psychological research, and recent evidence shows promise for the use of HCI and PSD principles. These principles initially show a positive effect in facilitating limit adherence, although this initial evidence needs to be expanded to include real-world trialling to support its overall efficacy. However, real-world testing of limit-setting tools that exist, appear to focus on the most intense sub-groups of gamblers. While justifiable by the fact that intense gamblers will be the group most likely in need tools to help them gain control over their gambling behaviour, the vast majority of gamblers play at safe levels, yet the effects of limit-setting on this group remain unclear.

It is evident that research concerning the setting of time limits has received less attention. While the one study identified here shows a positive result by demonstrating reduced gambling session length for those gamblers setting time limits, endorsement cannot be made using findings from a single study. Indeed, there is potential for maladaptive behaviour to occur when setting time limits. For example, potential unintended effects may include inadvertently causing gamblers to gamble larger sums of money to compensate for the shorter session duration they set themselves. Becauseof possible paradoxical, and unintended effects, full endorsement of the use of time limits cannot be made at the present time. A systematic and staged trial, encompassing a variety of gambling behaviour intensities, in which the effectiveness of limit-setting is monitored and evaluated over a sustained period of time appears to be the most advisable strategy moving forward before limit-setting receives full endorsement as a harm-minimisation tool.

In terms of actual behavioural evidence, results have shown that use of behavioural tracking tools that feedback to players the amount of time they have been gambling relative to normative data, show an overall reduced theoretical loss and gambling session duration. However, this effect is small with results from the *mentor* system showing its effect is only slightly (although significantly) above chance level. The use of colour coded feedback systems, informing players of their level of risk according to expressed gambling behaviour, appear to have a positive influence on a majority of gamblers in various sub-groups categorised according to their level of risk. Overall reductions in deposit limits have been found as a result of behavioural tracking systems for those gamblers already demonstrating safe and RG behaviour—an effect that is sustained at a six-month interval. While initial effects of behavioural tracking are not found for those players demonstrating a greater level of risk immediately following enrolment to such systems, positive effects begin to emerge at a six-month interval period, expressed in terms of reduced wagering and depositing, potentially indicating that behavioural tracking systems offer long-term benefits in the absence of immediate gains for more risky players. Evaluation of behaviour over a more sustained period of time should shed further light on this suggestion.

Unfortunately, the effects of behavioural tracking from the existing studies here either do not show a positive impact on the most risky gamblers, or such information cannot be extracted due to the methodological approach failing to distinguish problem gambling status of the participants. While attempts have been made to categorise risk levels according to expressed online gambling behaviour using algorithmic software, there is currently no consensus on how much this actually relates to external and more widely used screening measures of problem gambling behaviour. While positive evidence exists for the use of behavioural tracking systems as an RG tool, a future key issue involves determining which specific features of behavioural tracking tools are the most effective in facilitating and enabling a positive behavioural change in gamblers. It also needs to be ascertained if specific features are more effective according to the level of risk of the gambler, rather than assuming a one-size fits all approach.

A consistent limitation in much of the limit-setting and behavioural tracking research is that while there was generally a positive effect of the tools on reducing gambling behaviour, current research design limitations make it impossible to ascertain whether or not gamblers simply swap machines or gambling sites once their personal limits have been reached, or if the same applies as a way of avoiding negative behavioural feedback on behavioural tracking systems. It is not known how often this occurs, and epidemiological surveys may be required to ascertain if this is a concern for harm-minimisation research. One way around this, though arguably unlikely in the foreseeable future, is to have a centralised ‘hub’ whereby a player may gamble on multiple gambling sites but their overall expenditure, stake sizing, frequency and duration of play, and limit-setting function, is governed by a central system where all accounts held by a player all correspond to a unique identifier code. Thus, setting a limit on the central hub would mean that the personal limits applied as a maximum spend across all their gambling accounts.

Other harm-minimisation approaches, such as the use of note acceptor prohibition or modification have received less academic attention. However, note acceptor prohibition shows promise. Hansen and Rossow (2010) demonstrated a reduction in gambling frequency and problem gambling in a large sample if adolescent-aged gamblers as a result of note acceptor prohibition. These results were only applicable to one sub-group of gamblers (i.e., adolescents), though the effects were shown across a range of problem gambling severity levels.

## Conclusion

It is important to bear in mind the heterogeneous structural and situational characteristics across electronic gambling and online platforms, and the games themselves. Consequently, endorsing an RG tool fully requires testing it across a diverse range of game types. For example, tools effective in breaking dissociation in games with smaller stakes but rapid gameplay speeds, may not necessarily transfer to success in slower speed higher stake games. For this to happen, it is important to empirically investigate the psychological mechanisms of change that transfer a gambler from a cognisant state of control to a loss of self-control, according to specific gambling parameters, if indeed these mechanisms differ according to game types and their associated structural characteristics.

Results appear to support the notion that harm-minimisation tools should be viewed as a responsible gambling prevention measure for those who already gamble safely, or are at risk of developing a problem, rather than an intervention for those already exhibiting problem gambling behaviour. That said, non-gamblers or non-problem gamblers make up the majority of participants in all the studies outlined (compared to the numbers of problem gamblers). However, some studies did show some RG tool efficacy for high-intensity gamblers, although how this can be extended to apply to actual diagnostic measurements of problem gambling scores remains unanswered at present. A danger would be to assume that new tools and approaches being developed would not work for problem gambling sub-groups. However, problem gamblers should still be involved in the testing of new approaches so that opportunities are not missed with regards to assisting this group regain control of their gambling behaviour.

Whilst the limitations of laboratory-based experimental work are recognised, this does not expel their relevance in the research field of gambling harm-minimisation. Indeed, while ecological validity is largely lacking in such studies, they offer a level of experimental control often not afforded by real world research, allowing the impact of specific game manipulations and tools to be tested for both their positive and negative influences on behaviour and cognition. This is an important stage in the research process, as RG tools should demonstrate positive efficacy before being widely implemented in real-world settings, which may prove costly both financially and for the gamblers themselves if tools are capable of producing unintended effects. However, the progression from laboratory research to real-world application should not be linear. Where a better conceptualisation should be one of an iterative or cyclic relationship, with laboratory work paving the way for real world application, where then in turn, issues, observations, and ideas based on this real world application are fed back into the laboratory to allow next generation improvements to RG tools to be made.

Research in this field should remain both creative and flexible to both deal with potential changing landscapes of gambling, as well as to continue to strive for advancement of current harm-minimisation tool approaches. This creativity should also extend not only to advancing current ideas, for example, changing the content and layout of pop-up messaging to bring about greater cognitive and behavioural impact, but also continue to use science and psychological theory to develop new approaches yet to be investigated.

## Footnotes

1 Recent empirical evidence demonstrates that the delivery of PNF has clinical utility in that it reduces maladaptive gambling-related cognitions and behaviour amongst gamblers of various problem gambling severities (see Cunningham et al. 2009; Cunningham et al. 2012; Larimer et al. 2011; Neighbors et al. 2015). These studies were not included in the current review due to failure to meet within-session gambling harm-minimisation criteria, however, for a recent review of these studies see Marchica and Derevensky (2016).
